# Symptom effects and central mechanism of acupuncture in patients with functional gastrointestinal disorders: a systematic review based on fMRI studies

**DOI:** 10.1186/s12876-024-03124-y

**Published:** 2024-01-24

**Authors:** Lin Wang, Xiaoying Luo, Xiangli Qing, Shuangshuang Fang, Tianyuan Jiang, Qianying Wang, Zhuotai Zhong, Yang Yang, Jianqin Yang, Gengqing Song, Xiaolan Su, Wei Wei

**Affiliations:** 1 Department of Gastroenterology, Beijing Key Laboratory of Functional Gastrointestinal Disorders Diagnosis and Treatment of Traditional Chinese Medicine, Wangjing Hospital, China Academy of Chinese Medical Sciences, City, Beijing, China; 2 Dongzhimen Hospital, Beijing University of Chinese Medicine, Beijing, China; 3 Graduate School of Chengdu University of Chinese Medicine, Chengdu, China; 4 Department of Gastroenterology and Hepatology, MetroHealth Medical Center/Case Western Reserve University, Cleveland, OH USA

**Keywords:** Functional gastrointestinal disorders, Acupuncture, fMRI, Systematic review

## Abstract

**Background:**

Functional gastrointestinal disorders (FGIDs) are closely related to disorders of brain-gut interaction. FGIDs are the dominant disease of acupuncture treatment, which can improve the symptoms and emotional state.

**Aim:**

To evaluate the results and quality of the available clinical evidence and to summarize the central mechanism and effect of acupuncture on FGIDs.

**Methods:**

PubMed, EMBASE, Web of science, Cochrane Library, China National Knowledge Infrastructure (CNKI) were searched by computer to collect the randomized controlled trials (RCTs), which contained central mechanisms via fMRI research of acupuncture in the treatment of FGIDs patients. The search time limit was from the establishment of the database to June 22, 2022. Two researchers independently screened the literature, extracted data, and evaluated the quality.

**Results:**

Ten RCTs involving fMRI data were included in this study, including 4 Functional dyspepsia (FD) studies, 3 irritable bowel syndrome (IBS) studies, and 3 functional constipation (FC) studies. The score of improvements in both gastrointestinal symptoms and psychological symptoms showed that acupuncture could significantly improve the clinical symptoms of FGIDs patients, including abdominal pain, abdominal distension, frequency of defecation, and stool characteristics, and could relieve anxiety and depression symptoms of patients. Acupuncture could regulate brain functional connections and functional activity in FGIDs patients, mainly including insula, anterior cingulate cortex, prefrontal cortex, thalamus, hippocampus, amygdala and other brain regions.

**Conclusion:**

Acupuncture can improve gastrointestinal symptoms and psychological status in FGIDs patients, and regulate functional connectivity and activity of brain regions such as insula, ACC, PFC, thalamus, HIPP, amygdala, etc. These changes in brain activity may related to visceral sensation, pain regulation, emotion, but further studies of high quality are still necessary.

**Supplementary Information:**

The online version contains supplementary material available at 10.1186/s12876-024-03124-y.

## Introduction

Functional gastrointestinal disorders (FGIDs) are common clinical psychosomatic disorders of digestive dysfunction, which are characterized by chronic and recurrent gastrointestinal dysfunction without clinical organic lesions and lack of laboratory examination [1]. Functional dyspepsia (FD), irritable bowel syndrome (IBS), and functional constipation (FC) are the most common FGIDs in clinical practice. The prevalence of FD is about 11.5 to 14.5% globally [2] and as high as 23.5% in China [3]. The global prevalence rates of IBS and FC were 11.2 and 15.3%, respectively [4, 5]. Due to the difficult treatment of recurrent episodes, FGIDs bring a heavy burden to patients, society, and the medical and health system [6].

With the acceleration of life rhythm and the increase in work pressure, the role of psychological factors in the occurrence and development of FGIDs has attracted more and more attention. In 2016, the Rome Committee defined FGIDs as disorders of gut-brain interaction (DGBI) [7]. One study showed [8] that the incidence of anxiety and depression in FGIDs patients was 61.5 and 57.0%, and the gastrointestinal symptoms were positively correlated with anxiety and depression scores. The pathogenesis of FGIDs is complex and still not fully understood. However, it may be associated with disorders of gut-brain interaction, leading to gastrointestinal motility abnormalities, visceral hypersensitivity, intestinal microflora disorders, and mucosal immune function changes [6].

Patients with FGIDs are often accompanied by psychological disorders. The brain-gut axis refers to the bidirectional neural connection pathway between the digestive tract and the central nervous system; that is, exogenous or endogenous information can affect the sensory, motor, and secretory functions of the gastrointestinal tract through the central nervous system [9]. Dysfunction of the brain-gut axis is a major pathophysiological mechanism leading to the occurrence of FGIDs, and abnormalities of the brain-gut axis are one of the potential pathogenesis of FGIDs [6]. The development of neuroimaging techniques has contributed to the study of brain-gut axis damage in FGIDs [10]. The thalamus and primary somatosensory cortex (SI), and secondary somatosensory cortex (SII) of the brain are mainly associated with the first-order process of sensory information, while the prefrontal cortex(PFC), insula, and anterior cingulate cortex(ACC) are involved in the higher-order processes of cognitive assessment, attention, sensorimotor integration and emotional response [11, 12]. Using functional magnetic resonance imaging (fMRI) technology, studies have observed that compared with healthy people, the activation of ACC and amygdala, which are responsible for emotional and cognitive functions, is more obvious in the brain region of patients with FGIDs [13].

Acupuncture is a traditional external treatment in China, which has a long history of thousands of years and is widely used in the treatment of various diseases. In recent years, a number of clinical randomized controlled trials have demonstrated that acupuncture is effective and safe in the treatment of FGIDs [14–16]. It is now known that the mechanism of acupuncture is mainly related to the regulation of neural pathways, humoral immunity, serotonin pathway, and brain intestinal peptide secretion [17, 18], indicating the correlation between brain-gut interaction and acupuncture treatment of FGIDs. Brain functional imaging technology provides a visual and objective research approach to study the brain response mechanism of acupuncture treatment of FGIDs. Acupuncture along meridians can induce the response of specific brain regions in the central nervous system, which is one of the important scientific evidences that acupuncture regulates brain-gut interaction in the treatment of FGIDs. Studies have confirmed that acupuncture can improve the symptoms of abdominal pain, diarrhea, anxiety, and other symptoms in patients with IBS-D by affecting the functional connection between the hippocampus and the brain regions related to emotion and visceral sensation [19]. Basolateral amygdala, hippocampus and medial prefrontal cortex (mPFC) may be the target brain regions for the central response to acupuncture treatment of functional dyspepsia [20–22]. In recent years, clinical randomized controlled trials (RCTs) involving acupuncture on brain function changes of FGIDs patients have gradually emerged. However, the types of acupuncture and acupoint selection vary among studies, which makes it confusing to evaluate the efficacy of acupuncture on FGIDs and to understand the central mechanism.

Therefore, in order to better understand the central mechanism and efficacy of acupuncture in the treatment of FGIDs patients, our study summarizes and evaluates relevant clinical controlled trials as comprehensively as possible, aiming to conduct a comprehensive investigation of the core brain regions related to acupuncture treatment of FGIDs.

## Methods

This study has been registered on PROSPERO (https://www.crd.york.ac.uk/PROSPERO). The registration number is CRD42022342512.

### Search strategy

This study used a systematic search strategy that followed the PRISMA guidelines. Electronic searches were conducted in five databases, including PubMed, EMBASE, Web of science, Cochrane Library, China National Knowledge Infrastructure (CNKI). The retrieval time was completed in June 22, 2022. Medical Subject Headings (MeSH) included “acupuncture”, “Gastrointestinal Diseases”, “Irritable Bowel Syndrome”, “functional dyspepsia”, “functional constipation”, “Magnetic Resonance Imaging”, “randomized controlled trial”. A complete search strategy was attached in a supplementary document (Supplementary Document 1).

### Study selection

#### Inclusion criteria

#### Study type

Clinical randomized controlled trials (RCTs) were published in Chinese or English. All RCTs should state the specific method of randomization or refer to “random,” which does not restrict the implementation of blinding.

#### Participants

The inclusion criteria were adult (18–75 years old) diagnosed with FD, IBS, or FC, according to Rome I ~ IV criteria, regardless of gender or race.

#### Intervention measures

Treatment group: The intervention in the treatment group was acupuncture (mainly consist of manual acupuncture (MA), electroacupuncture (EA), and transcutaneous electrostimulation (TEA); exclude moxibustion, acupoint catgut embedding or acupoint injection), regardless of the number of acupoints or the duration of treatment.

Control group: The intervention in the control group was sham acupuncture, moxibustion, medication, or no intervention. The sham acupuncture and moxibustion should operate on the same acupoints as the treatment group.

#### Outcome evaluation indicators

1. Main outcome measure: Brain functional magnetic resonance imaging (fMRI) data.
2. Secondary outcome indexes are clinical symptom score, anxiety and depression score.The original data was complete and extractable.

#### Exclusion criteria

(1)studies that did not meet the aforementioned inclusion criteria; (2)Unpublished original materials; (3)Unable to obtain full text or incomplete data; (4)Interventions are influenced by other treatments; (5)Duplicate published literatures; (6)Non-clinical RCTs.

### Data extraction

According to inclusion criteria and exclusion criteria, two researchers independently read titles, abstracts and full texts for literature screening. Data were extracted, including author, year of publication, sample size and gender of participants, diagnostic criteria, interventions (acupoint selection, treatment duration, and frequency), primary efficacy evaluation, imaging modality/condition, and results of brain imaging data. If there is any objection, a third party should be involved in the evaluation.

### The quality assessment of included literatures

The quality of all included literatures was assessed independently by two researchers, and if there was any objection, the third party would evaluate it. This study used RevMan 5.3 software based on *Cochrane Handbook for Systematic Reviews* to assess the risk of bias of included literatures, including random sequence generation, allocation concealment, blinding, incomplete outcome data, selective reporting, and other biases. According to the performance of the included literatures in the above evaluation items, the judgment of low risk, unclear risk and high risk were given, respectively.

### Statistical analysis

Review Manager 5.3 was used to analyze the merge effect. Continuous variables were analyzed with mean difference (MD) and expressed with 95% confidence interval (CI). The χ^2^ test and the inconsistency index statistic I^2^ were used to statistically test the heterogeneity. If the heterogeneity was significant (I^2^ > 50% or *P* < 0.1), a random effect model was used. Otherwise (I^2^ ≤ 50% or *P* ≥ 0.1), a fixed effect model was used. If the included studies were highly heterogeneous, subgroup analysis should be performed to identify potential sources of heterogeneity.

## Results

### Retrieval results

**Fig. 1:**
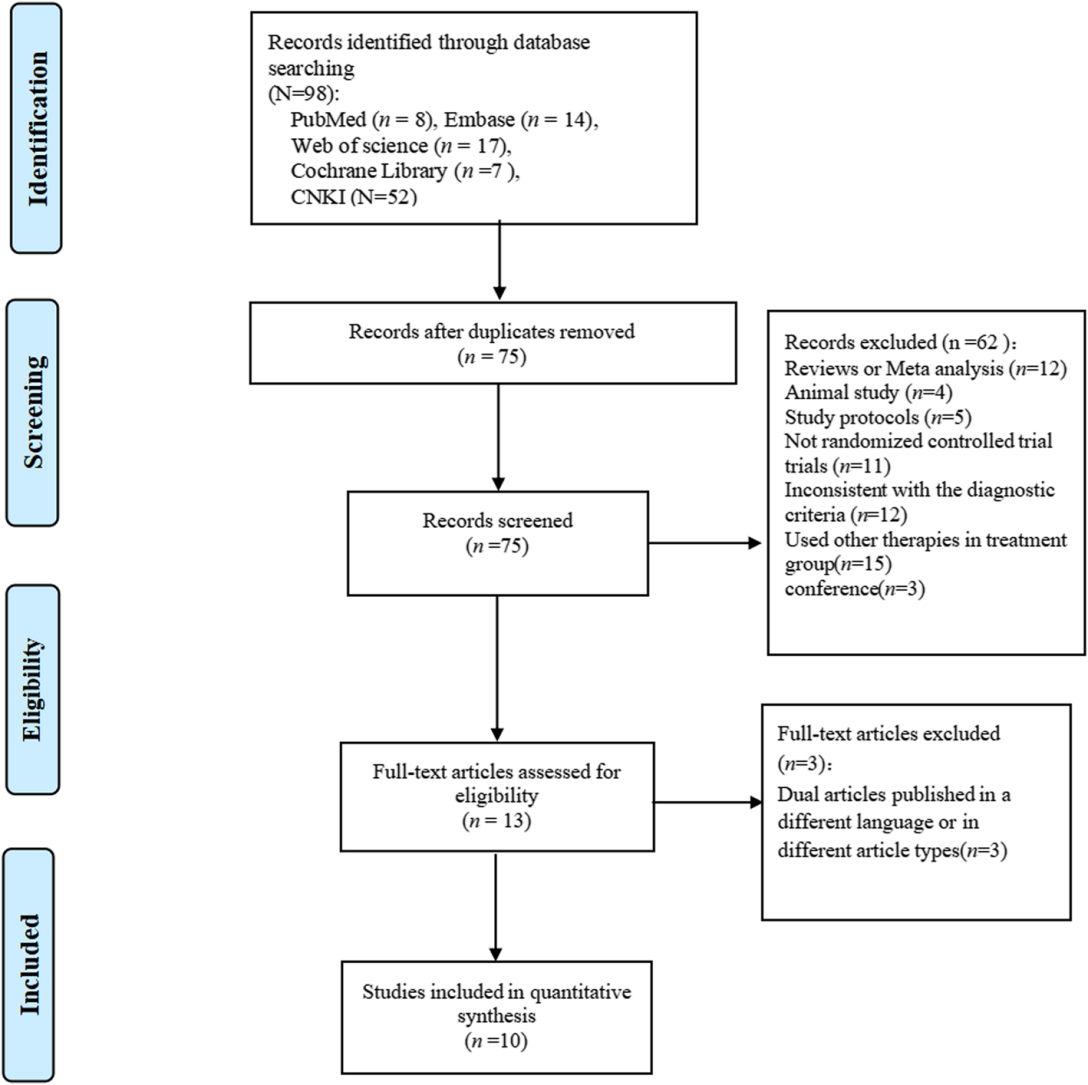
Article screening process

According to the literature search strategy, 98 articles were initially retrieved from five databases, including 8 articles in PubMed, 14 articles in EMBASE, 7 articles in Cochrane, 17 articles in Web of Science and 52 articles in CNKI. Second, a total of 65 articles were excluded by reading article titles, abstracts, and full texts. Two of the articles [23, 24] were the same study, but included different fMRI data. Ultimately, we included 10 studies (including 11 articles) [21, 23–32]. The process of literature screening based on PRISMA is shown in Fig. 1.

### Study characteristics

This study ultimately included 10 RCTs, including 4 FD studies [21, 23–26], 3 IBS studies [27–29], and 3 FC studies [30–32]. All studies used the Roman diagnostic criteria, published from 2012 to 2021, and included a total of 474 participants, 406 of whom completed fMRI. All of the studies were from China, and six of them [21, 25–29] were published in English. The proportion of females in these participants was 10 to 97.1%, except for two studies not mentioned [27, 29]. The intervention measures in the treatment groups were acupuncture, including MA, EA and TEA. Six of the studies used sham acupuncture (SA) [21, 23, 24, 26, 28] or waiting for treatment [31, 32] as controls, and three used moxibustion [25, 27, 29] or medication (polyethylene glycol) as controls [30]. All studies reported the results of brain fMRI, of which three IBS [27–29] studies used event-related fMRI, and the rest used resting state fMRI. The acupuncture efficacy in FGIDs was reflected with certain gastrointestinal symptom scales in eight studies [21, 23, 24, 26, 27, 29–32], and anxiety and depression rating scales were put to use in five studies [21, 23, 24, 26, 27, 29] to assess psychological status. Detailed information of the included study characteristics is shown in Table 1.

**Table 1:** Characteristics of included studies

Reference	Language	Country	Diagnostic criteria	Sample size(T:C)	fMRI Sample size(T:C)	Sex ratio (male: female)	Interventions		Duration and frequency	Clinical outcomes
							T	C		
**FD**										
SUN R etal,2021 [21]	English	China	Rome III	64(33:31)	41(21:20)	64(8:56)	MA(CV12, ST36, deqi)	SA(CV12, ST36, without deqi)	T&C:once a day, five times per week, for 4 weeks	NDSI, SAS, SDS
Zhou2020: Chen etal,2021 [23]	Chinese	China	Rome IV	54(26:28)	54(26:28)	54(13:41)	taVNS(In the concha cavity)	sham-taVNS(upper ear navicular)	T&C:15 min, twice per day, for 2 weeks	overall symptoms of FD, NDI, HAMA, HAMD, SAS, SDS
Li etal,2021 [25]	English	China	Rome IV	56(29:27)	56(29:27)	56(32:24)	MA(ST36)	MOX(ST36)	T&C:once a day, five times per week, for 4 weeks	NR
Sun etal,2021 [26]	English	China	Rome III	32(16:16)	32(16:16)	32(6:26)	MA(CV12, ST36, deqi)	SA(CV12, ST36, without deqi)	T&C:once a day, five times per week for 4 weeks	NDSI, NDI-QOL, SAS, SDS
**IBS**										
Zhao etal, 2018 [27]	English	China	Rome III	60(30:30)	60(30:30)	NR	EA(ST25,ST37)	Mox(ST25,ST37)	T&C:30 min each time, 6 times per week,for 4 weeks	VAS, HAMA, HAMD
Chu etal,2012 [28]	English	China	Rome III	30(15:15)	30(15:15)	30(15:15)	EA(ST36,ST37,SP6)	SA(ST36,ST37,SP6;The needle tips were hidden inside foam cubes)	30 min	NR
Zhao etal, 2015 [29]	English	China	Rome III	60(30:30)	15(7:8)	NR	EA(ST25,ST37)	Mox(ST25,ST37)	T&C:30 min each time, 6 times per week,for 4 weeks	VAS-IBS, HAMA, HAMD
**FC**										
Chen, 2020 [30]	Chinese	China	Rome IV	34(17:17)	34(17:17)	34(1:33)	EA(ST36,ST37,ST25)	PEG	T:once a day, five times per week for 2 weeks; C:once a day, once a packet for 10 g, five times per week for 2 weeks	PAC-SYM, SAS, SDS
Hou, 2020 [31]	Chinese	China	Rome IV	34(17:17)	34(17:17)	34(3:31)	EA(ST36,ST37,ST25)	WT	T:once a day, five times per week for 2 weeks	PAC-SYM, PAC-QOL, SAS, SDS
Ma, 2021 [32]	Chinese	China	Rome IV	50(32:18)	50(32:18)	50(45:5)	MA(ST25, ST36, ST37, non-acupoints)	WT	T:once a day, five times per week for 2 weeks	PAC-SYM, PAC-QOL

*taVNS* transcutaneous auricular vagus nerve stimulation, *sham-taVNS* auricular acupoints with non-vagus nerve, *MA* manual acupuncture, *MOX* moxibustion, *EA* Electroacupuncture, *SA* sham acupuncture, *WT* waiting treatment, *PEG* Polyethylene glycol, *CV12* Zhongwan, *ST36* Zusanli, *ST25* Tianshu, *ST37* Shangjuxu, *SP6* Sanyinjiao

### Clinical outcomes

All studies reported brain imaging data results, and eight [21, 23, 24, 26, 27, 29–32] of the studies reported clinical outcomes, which were divided into two categories: FGIDs symptoms and psychological outcomes.

#### FGIDs symptoms outcomes

A total of 8 studies compared the FGIDs symptoms of patients after acupuncture treatment, including 3 FD studies [21, 23, 24, 26], 2 IBS studies [27, 29], and 3 FC studies [30–32]. Combined results showed that acupuncture could significantly improve the clinical symptoms of FGIDs patients, including abdominal pain, abdominal distension, frequency of defecation, and stool characteristics, etc.

##### FD symptom outcomes

In FD clinical trials, the most commonly used FD symptom assessment questionnaire is the NDI questionnaire [33], including the Nepalese Dyspepsia Symptom Index (NDSI) [34] and the Nepalese Dyspepsia Life Quality Iindex (NDLQI), which can record both FD symptoms and FD specific quality of life. In this systematic review, two studies used the NDI questionnaire [23, 24, 26], one study [21] used the NDSI score to evaluate clinical symptoms of FD.

The taVNS group could significantly improve the overall symptoms of FD and reduce the NDI score (both *P* < 0.05), while the sham-taVNS group only improved the overall symptoms [23, 24]. Our review showed that the clinical symptoms of FD improved after both acupuncture and sham acupuncture treatment (P < 0.05), and the MA *Deqi* group showed greater decrease of the FD symptom score than that in the MA without *Deqi* group (*P* < 0.05) [21, 26].

##### IBS symptom outcomes

Both IBS studies used Visual Analogue Scale (VAS) to assess major gastrointestinal symptoms, one in patients with IBS-C [27] and the other in patients with IBS-D [29]. Our results showed that after 4 weeks of treatment in IBS-C patients, the improvements in abdominal distension, defecation frequency, difficulty in defecation, and stool form were significantly greater in the EA group than in the MOX group (*P* < 0.01) [27]. After 4 weeks of treatment in IBS-D patients, compared with before treatment, the EA group reported significant improvements in abdominal pain, abdominal distension, defecation emergency and defecation frequency (*P* < 0.05 or *P* < 0.01), but the MOX group showed more significant improvements in emergency defecation and defecation frequency (*P* < 0.01) [29].

##### FC symptom outcomes

One of the results showed [30] that the PAC-SYM scores of the two groups (acupuncture vs. medication) of FC patients after treatment were significantly decreased (*p* < 0.01), while there was no statistical difference in the improvement of the PAC-SYM scores between the two groups (*p* > 0.05). Two other studies [31, 32] showed that the PAC-SYM and PAC-QOL (Patient Assessment of Constipation Quality of Life) scores of FC patients were significantly decreased after acupuncture treatment (*P* < 0.01 or *P* < 0.05). Among them, in Hou’s study [31], the improvement values of PAC-SYM and PAC-QOL scores in the acupuncture group were greater than those in the WT group (*P* < 0.05), while in Ma’s study [32], only the improvement in PAC-QOL score was greater than that in the WT group (*P* < 0.05).

**Fig. 2:**
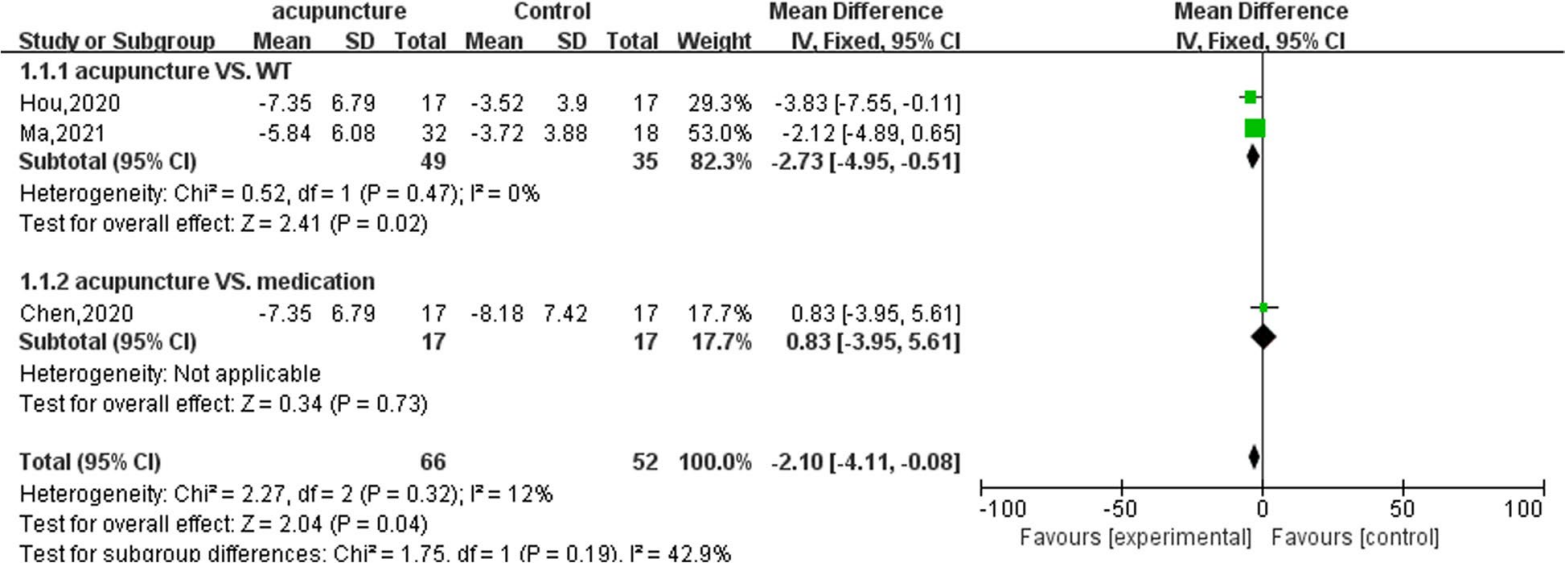
Meta-analysis of PAC-SYM of treatment group and control group

The comprehensive results of the meta-analysis showed that acupuncture treatment could effectively reduce the PAC-SYM score in patients with FC. [MD = − 2.10; 95%CI (− 4.11, − 0.08); *Z* = 2.04; *p* = 0.04]. Subgroup analysis showed that compared with the WT group, acupuncture could reduce the PAC-SYM score of FC patients [31, 32] [MD = − 2.73; 95%CI (− 4.95, − 0.51); *Z* = 2.41; *p* = 0.02], but there was no significant difference compared with the medication group [30] [MD = 0.83; 95%CI (− 3.95, 5.61); *Z* = 0.34;*p* = 0.73] (Fig. 2).

#### Psychological outcomes

Patients with FGIDs are often accompanied by psychological disorders. Therefore, anxiety and depression scores were included as secondary outcomes in this study. A total of 5 studies compared the psychological outcomes of patients with FGIDs after acupuncture treatment, including 3 FD studies [21, 23, 24, 26], 2 IBS studies [27, 29]. In our systematic review, Zung Self Rating Anxiety Scale (SAS) and Zung Self Rating Depression Scale (SDS) [21, 23, 26], Hamilton Anxiety Rating Scale (HAMA) and Hamilton Depression Rating Scale (HAMD) [23, 24, 27, 29] were used to evaluate psychological status. Combined results showed that acupuncture could significantly relieve anxiety and depression in FGIDs patients.

In FD patients [21, 23, 24, 26], both the acupuncture group and the sham acupuncture group had significantly decreased anxiety and depression scores after treatment (*P* < 0.05).Three studies [21, 23, 26] including 150 participants compared the SAS and SDS scores after acupuncture treatment in patients with FD. The heterogeneity test showed high heterogeneity (I^2^ = 98%, *p* < 0.00001; I^2^ = 86%, *p* = 0.0007), the randomized effect model was adopted.

**Fig. 3:**
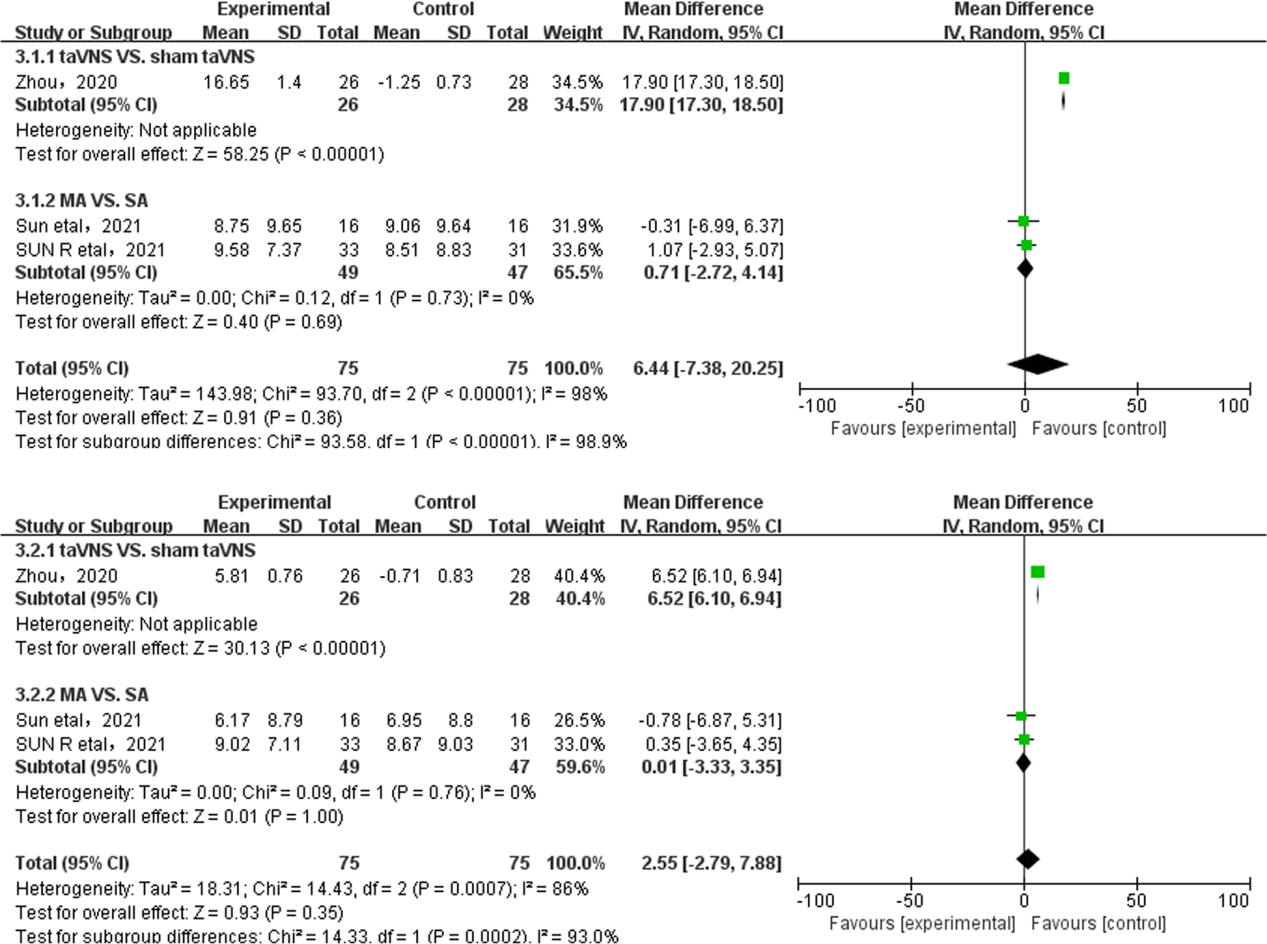
Meta-analysis of SAS/SDS of treatment group and control group

The comprehensive results of the meta-analysis showed that there was no significant difference in SAS and SDS scores between the two groups in patients with FD [MD = 6.44; 95%CI (− 7.38, 20.25); Z = 0.91; *p* = 0.36]; [MD = 2.55; 95%CI (− 2.79, 7.88); Z = 0.93; *p* = 0.35]. Subgroup analysis showed that compared with the sham-taVNS group, taVNS could reduce the SAS and SDS score of FD patients [23] [MD = 17.90; 95%CI (17.30, 18.50); Z = 58.25; *p* < 0.00001]; [MD = 6.52; 95%CI (6.10, 6.94); Z = 30.13; *p* < 0.00001], but there was no significant difference between the MA group and the SA group [21, 26] [MD = 0.71; 95%CI (− 2.72, 4.14); Z = 0.40; *p* = 0.69]; [MD = 0.01; 95%CI (− 3.33, 3.35); Z = 0.01; *p* = 1.00](Fig. 3).

The IBS clinical studies included in this review all used moxibustion as the control group. IBS-C patients in the EA and MOX groups had significantly improved anxiety and depressive after treatment (*P* < 0.01) [27], and the improvements were significantly higher in the EA group than in the MOX group (*P* < 0.01), whereas in the IBS-D study, MOX treatment was more effective than EA treatment (*P* < 0.01) [29].

### Central mechanism-brain imaging data

Acupuncture-related brain activities can be divided into four categories for summary and analysis: comparison before and after acupuncture treatment, comparison between acupuncture and sham acupuncture or no intervention groups, comparison between acupuncture and moxibustion groups, comparison between acupuncture and medication groups. The activation of the most frequently reported brain regions (temporal gyrus, insula, ACC, PFC, thalamus, HIPP, amygdala, posterior cingulate, etc) are summarized in Table 2.

**Table 2:** Brain imaging data of frequently reported brain areas

	T after treatment VS Baseline		T VS C	
	Resting state	Event-related((CRD) stimulation)	Resting state	Event-related((CRD) stimulation)
HIPP	(left)ALFF^a^ [23],(Right)ALFF^a^ [25, 30],(lateral)ALFF^a^ [25],(left)FC with insula^a^ [32]		(left)ALFF^c^ [23],(left)FC with inferior frontal gyrus^c^ [32],	
Temporal Gyrus	(middle)Reho^a^ [24],(middle)ALFF^b^ [30],(superior)ALFF^b^ [30],(superior)DMN FC^a^ [31]		(inferior)ALFF^c^ [23],(superior)ALFF^c^ [23],(middle)ALFF^c^ [23]	
PrG	(Left)Reho^a^ [24]			
BLA	(left)rsFC with right cuneus^a^ [21],rsFC with right HIPP and INS/putamen^b^ [21],(right)with left PCC, right HIPP and INS/putame^b^ [21]		(left)rsFC with bilateral INS, putamen and MCC/PCC, HIPP^d^ [21]	
ACC	rsFC with right SMA and bilateral PrG/Po^a^ [26], rsFC with right precuneus, PCC, HIPP/paraHIPP^b^ [26], (left)ALFF^a^ [30]	activation voxel value^b^ [27],brain activation^a^ [28]	(right)rsFC with right cerebellum^c^ [26]	
IC		activation voxel value^b^ [27]		
PFC		activation voxel value^b^ [27, 29], brain activation^a^ [28]		
Insula	(Right)ALFF^a^ [30]		(left)ALFF^c^ [23]	(right)activation^c^ [28]
PCG	(left)DMN FC^a^ [31]		(left)DMN FC↑* [29]	
Nucleus			(right)fALFF^c^ [25]	
Occipital Gyrus			(middle)fALFF^d^ [25]	
PCC			(right)ALFF^c^ [30]	
Thalamus		activation^a^ [28]		activation^c^ [28]

*T* treatment group, *C* control group, *HIPP* hippocampuspara, *HIPP* parahippocampal, *PrG* precentral gyrus, *BLA* basolateral amygdala, *ACC* anterior cingulate cortex, *IC* insular cortex, *INS* Insular, *PFC* prefrontal cortex, *CRD* colorectal dilatation, *PCG* Posterior Cingulate Gyrus, *PCC* posterior cingulate cortex, *MCC* middle cingulate cortex, *PoG* postcentral gyrus, *DMN* Default Mode Network, *FC* functional constipation, *fALFF/ALFF* fractional amplitude of low frequency fluctuations/amplitude of low frequency fluctuation, *rsFC* resting-state functional connectivity; ^a^, greater than baseline; ^b^, lower than baseline; ^c^, greater than control group; ^d^, lower than control group

#### Comparison before and after acupuncture treatment

##### Resting state fMRI before and after acupuncture treatment

After taVNS treatment for 2 weeks, the ALFF values in the left HIPP and left paraHIPP of the FD patients increased (*P* < 0.05) [23], the regional homogeneity (ReHo) values in the left middle temporal gyrus and left precentral gyrus (PrG) increased (*P* < 0.05) [24]. After 4 weeks of Zusanli (ST36) acupuncture treatment, the fALFF values in the right paraHIPP, left cerebellum, and left paraHIPP in FD patients were significantly increased (*P* < 0.01), and the fALFF values of the right middle occipital gyrus, right cuneus, left precuneus, left middle occipital gyrus, right superior frontal gyrus, left superior frontal gyrus, left inferior frontal gyrus, and the left superior frontal gyrus were significantly decreased (*P* < 0.01) [25]. After treatment of FD patients with acupuncture *Deqi*, the left BLA rsFC with right cuneus increased significantly, the left BLA rsFC with right HIPP and bilateral INS/putamen decreased significantly, the right BLA rsFC with left PCC, right HIPP and bilateral INS/putamen decreased significantly [21]. The acupuncture with *Deqi* group showed rsFC increased in the ACC subregion of the right supplementary motor area (SMA) and bilateral PrG/postcentral gyrus (PoG) in FD patients, while rsFC in the right precuneus, middle occipital gyrus, bilateral PCC, HIPP/paraHIPP, angular gyrus and superior parietal lobule (SPL) decreased after 4 weeks treatment [26].

After 2 weeks of MA treatment in FC patients, the ALFF values of brain regions in the Default Mode Network (DMN), such as right HIPP and left superior parietal gyrus, and the Salience Network (SN), such as the right anterior insula and left ACC increased. While the ALFF values in the DMN regions, such as left superior parietal gyrus, right superior temporal gyrus, left middle temporal gyrus and left supramarginal gyrus decreased [30]. Two weeks after treatment, the MA group showed that the functional connectivity of the left PCC (IC2), left precuneus (IC5), right superior temporal gyrus (IC25) and right cuneus (IC26) within the DMN was enhanced [31]. After MA treatment in patients with FC, the functional connectivity between the left HIPP and the right inferior frontal gyrus triangle, bilateral insula, and right inferior frontal gyrus was enhanced [32].

##### Event-related fMRI before and after acupuncture treatment

After 4 weeks of EA treatment, the activation voxel values of ACC, right insular cortex (IC) and PFC brain regions decreased in IBS-C patients under 150 ml colorectal dilatation (CRD) stimulation [27], while only the activation voxel values of PFC brain regions decreased in IBS-D patients under 150 ml CRD stimulation [29]. During (15 min) and immediately (30 min) after EA, the brain activation of ACC, bilateral PFC, thalamus, temporal regions and right insula increased [28].

The inconsistent results of these several studies may be due to different disease types (FD or IBS or FC) of FGIDs patients, intervention measures (MA or EA or taVNS), acupuncture points, and the duration or number of treatments.

#### Comparison between acupuncture and sham acupuncture or no intervention groups

Compared with the sham-taVNS control group, the ALFF values of the left inferior temporal gyrus, left paraHIPP, left HIPP, left orbital inferior frontal gyrus, left insula, left superior temporal gyrus temporal pole, left fusiform gyrus and left middle gyrus temporal pole in the taVNS treatment group of FD patients increased (*P* < 0.05) [23]. Compared with the group without obvious *Deqi*, the left BLA rsFC with bilateral INS, putamen and middle/posterior cingulate cortex (MCC/PCC), right pallidum and HIPP in the obvious *Deqi* group of FD patients decreased significantly after treatment [21]. Compared to the actual *Deqi* group, the actual without *Deqi* group showed significantly increased right 17(ACC subregion) rsFC with right cerebellum after treatment.There was no significant difference in left 17 and bilateral S2 rsFC between the two groups of FD patients after treatment [26]. Compared with sham acupuncture, the activation of the right insula, pulvinar and medial nucleus of the thalamus in IBS patients in the EA group was significantly increased [28]. Compared to the WT group, FC patients in MA group showed increased functional connectivity in the left PCG (IC2) and right precuneus (IC25), and decrease in the left supramarginal gyrus (IC5) and left cuneus (IC26) after treatment [31]. After treatment of FC patients, the functional connectivity of left HIPP and inferior frontal gyrus of MA Group were greater than that in WT Group [32].

#### Comparison between acupuncture and moxibustion groups

Compared with acupuncture and moxibustion after Zusanli (ST36) treatment of FD patients, the brain area with acupuncture higher than the fALFF value of moxibustion was mainly right putamen (*P* < 0.01), and the brain area with lower fALFF value was mainly mid-occipital gyrus (*P* < 0.01) [25]. In the study of Chu et al [27] and Zhao et al [29], there was no significant difference in the activity of ACC, left IC,right IC, and PFC between the EA group and the MOX group, regardless of whether they were IBS-C or IBS -D patients(*P*>0.05).

#### Comparison between acupuncture and medication groups

Compared with Polyethylene glycol (PEG) treatment, the ALFF value of left PCC in FC patients was significantly increased after MA treatment [30].

### Methodological quality

**Fig. 4:**
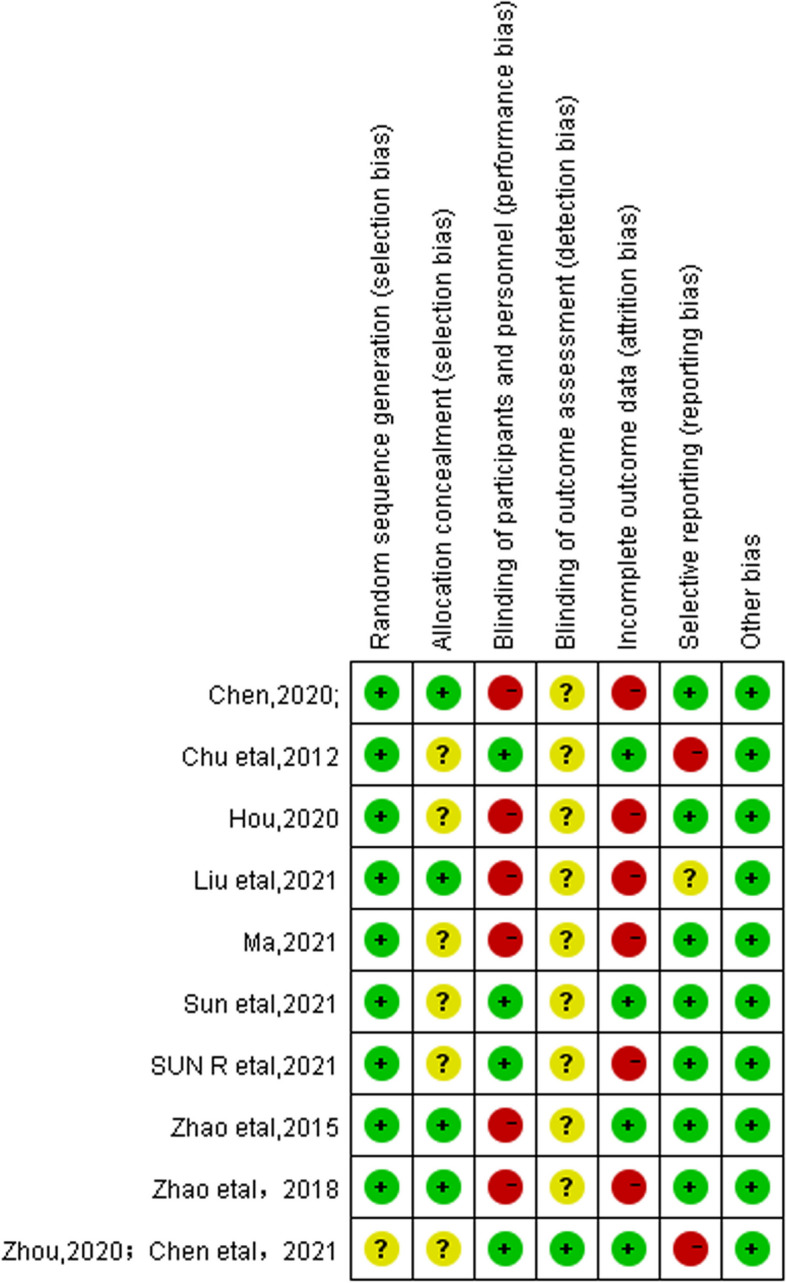
Assessment of the risk of bias of each included trial Green: Low risk of bias; Yellow: Unclear risk of bias: Red: High risk of bias

The risk of bias and methodological quality assessment of the included studies are shown in Fig. 4. (1) Selection bias: All included studies were RCTs, of which 8 studies [21, 25–32] used the “random number table” or “computer-generated random” method to generate random sequences, and only one study [23, 24] mentioned the word “random”, so it was evaluated as “unclear”. The allocation concealment method of four studies [25, 27, 29, 30] was sealed in envelopes, and the remaining six studies [21, 23, 24, 26, 28, 31, 32] did not mention the specific concealment method. (2) Performance bias: A proportion of studies [25, 27, 29–32] used non-acupuncture controls, such as moxibustion or oral medication, which can clearly detect the treatment method and judged as “high risk”. (3) Detection bias: Only one study [23, 24] showed that doctors, subjects, data collectors, and data analysts were all blinded, and none of the other studies described whether blinding was used in the evaluation of outcome indicators, so they were judged as “unclear”. (4) Attrition bias: Six studies [21, 25, 27, 30–32] reported case dropout and specific reasons for dropout, but no intentionality analysis was performed when reporting the outcome, so it was judged as “high risk”. (5) Reporting bias: Two studies [23, 24, 28] did not mention clinical trial registration, so they were judged as “high risk”. One study [25] did not provide exact clinical trial registration information, so it was judged as “unclear”. (6) Other bias: All studies found no significant other biases.

## Discussion

Acupuncture has been widely used in the treatment of FGIDs [35, 36], and has achieved good therapeutic effects, mainly in IBS, FD, FC [15, 17]. Numerous clinical studies have shown that acupuncture can significantly improve the symptoms of epigastric pain, early satiety, and epigastric burning in patients with FD [37], regulate gastric motility [38], and alleviate anxiety and depression [36, 38]. Acupuncture has a significant clinical effect on IBS-D, which can reduce the visceral sensitivity of IBS-D patients [39] and regulate the structure and diversity of intestinal microbiota in IBS-D patients [40]. Acupuncture can effectively relieve the clinical symptoms of FC patients [41], improve the frequency of defecation and quality of life [42, 43]. These effects may be related to the regulation of sympathetic and parasympathetic nerve functions by acupuncture to promote human balance.

Acupuncture point is the initial response site of acupuncture, and also the source link of acupuncture effect [44]. As the “trigger” of acupuncture regulation and the basic functional unit of the body communication, response and regulation, acupuncture points play an important role in the transmission and regulation of the body substance, energy and information [45]. ST36, CV12, ST25, ST37, PC6 are commonly used acupoints in the treatment of FGIDs, which have achieved good clinical effects [14, 46, 47]. Transcutaneous auricular vagus nerve stimulation (taVNS) is a modern scientific therapy that integrates traditional Chinese auricular acupoint theory and vagus nerve regulation. Clinical studies have shown that taVNS can relieve the symptoms, improve the quality of life, and solve the psychological problems of FD patients [23, 24, 48]. Because taVNS can provide non-invasive treatment and is more easily accepted by patients, it can be used as a new approach for acupuncture treatment of FGIDs. Recent studies have shown that abnormal brain-gut interaction is an important cause of FGIDs [6, 13, 49], and the regulation of acupuncture on the brain-gut axis is the main focus of its treatment of FGIDs [49, 50]. With the development of functional brain imaging techniques, more and more studies have been conducted on the correlation between FGIDs and central nervous system. However, the current researches on the central mechanism of acupuncture based on high-quality clinical trials related to FGIDs are still insufficient. The main purpose of this study is to summarize and reveal the potential central mechanisms of acupuncture in the treatment of FGIDs based on current published clinical trials.

A total of 10 studies were included in this systematic review and acupuncture-related functional brain activity (resting state, event-related) and clinical outcomes were measured. fMRI studies found that due to differences in acupoints and acupuncture methods, the brain effects of acupuncture have both commonalities and differences [51]. In our review, the brain regions responding to acupuncture of FGIDs were frequently found in temporal gyrus, insula, ACC, PFC, thalamus, HIPP, amygdala, posterior cingulate, etc. When compared to before acupuncture treatment, FGIDs patients showed greater activation in the HIPP, amygdala, cingulate gyrus, and temporal gyrus in the resting state, as well as altered activation in the insula, prefrontal cortex, anterior cingulate gyrus, and thalamus during the CRD stimulation. In addition, the results of our study showed that the clinical symptom changes of FGIDs patients before and after acupuncture treatment were correlated with fMRI data. The study by Sun et al [21] showed that the changed of NDSI score (before and after) of FD patients in acupuncture *Deqi* group was significantly positively correlated with their Fisher’s transformed z value of left BLA rsFC with right HIPP (*r* = 0.394, *p* = 0.011), but significantly negatively correlated with rsFC between right centromedial amygdala (CMA) and left PFC(r = − 0.463, *p* = 0.035). And the changed NDI QOL score was significantly positively correlated with the altered rsFC between amygdala subregions and right SPL (*r* = − 0.597, *p* = 0.04) [26]. The changed of functional connectivity in the left angular gyrus of FC patients before and after acupuncture treatment were positively correlated with the improvement of PAC-SYM and PAC-QOL (*r* = 0.598, *p* = 0.011) [31], and the changed of ALFF values in the left parietal lobule were significantly positively correlated with the improvement of CCS score (*r* = 0.626, *p* = 0.007) [30]. In Ma’s study [32], the changed of functional connectivity between the left HIPP and the left insula was positively correlated with the improvement value of PAC-SYM (*r* = 0.404, *p* < 0.022) and PAC-QOL score (*r* = 0.404, *p* < 0.022) in the acupuncture group before and after treatment.

The development of chronic pain is associated with synaptic plasticity and changes in the CNS and various neural regions that regulate pain. Chronic pain causes structural and functional changes in cortical limbic brain regions. These changes can induce negative emotional states, underpinned by common neuroplasticity changes in chronic pain and negative emotional states [52]. The gastrointestinal symptoms of patients with FGIDs are related to the central nervous system (CNS) through the enteric nervous system (ENS). Long-term abnormal nerve signals change the function or structure of the CNS, and then abnormally regulate gastrointestinal motility and sensory functions [6, 7]. Brain imaging studies have revealed that FGIDs have abnormalities in brain structure and function. In a study [53], CiteSpace software was used to summarize and analyze the existing literatures on the central pathological changes of FGIDs based on MRI technology, and the results showed that ACC, PFC and DMN were the key brain regions affecting FGIDs. At present, a large number of studies have shown that the brain regions related to gastrointestinal regulation, cognition and emotion are the main brain regions responding to acupuncture in the treatment of FGIDs [54]. In the results of our review, temporal gyrus, insula, ACC, PFC, thalamus, HIPP, amygdala, and posterior cingulate were the key brain regions of acupuncture in the treatment of FGIDs.

Imaging studies have found that the prefrontal cortex (PFC) and extensive limbic systems are involved in brain-gut interaction process [55, 56]. And our study summarized the common brain regions of acupuncture in the treatment of FGIDs. PFC is an advanced center for pain perception, which integrates information from peripheral, cognitive regulation of pain, and evaluates or responds to emotional aspect of pain sensation [10, 57]. In FGIDs patients with negative emotions caused by long-term symptoms, abnormal signals from the peripheral nervous system are transmitted to the PFC brain region, and the PFC brain region coordinates and integrates the information. Long-term abnormal signals may lead to abnormal brain structure and function [53]. In the studies of FGIDs such as IBS, FD and FC, the function and structure of PFC have been shown to be abnormal [5, 58, 59]. Our review showed that the activity of the PFC was altered during CRD stimulation after acupuncture treatment [27–29]. And it was also observed that there was a correlation between the changes in clinical symptoms and PFC activity before and after acupuncture treatment in patients with FGIDs [21].

Brain regions related to the limbic system (insula, cingulate gyrus, amygdala, HIPP, paraHIPP, thalamus, etc.) are involved in the formation and expression of “pain-emotion-cognition”, which can regulate visceral activities and affect emotions [18, 60]. ACC is the anterior part of cingulate gyrus, which is particularly important for the processing of emotions, gastrointestinal sensory signals and other information [61, 62]. Visceral hypersensitivity is the main pathophysiological mechanism of FGIDs. A growing number of researches have found that the ACC in the CNS is associated with visceral hypersensitivity induced by mental stress [63]. Mental stress can enhance ACC activity, and the increased activation of ACC can lead to the related symptoms caused by visceral hypersensitivity, such as abdominal pain, diarrhea, abdominal distension and so on. fMRI studies have found that after FGIDs patients receive CRD stimulation, multiple brain regions (such as ACC) showed significant activation of different intensities [56]. This means that the abnormal activity of ACC may be related to the chronic visceral sensory and psychological state of patients with FGIDs.

The insula and thalamus are components of the visceral sensory nerve center in the brain [64]. The insula is involved in a variety of sensory and cognitive processes, such as learning, memory, and sensory perception [65, 66]. In recent years, with the in-depth study of insular cortex, it has been found that the insular cortex is closely related to emotion, homeostatic function, chronic pain, and hyperalgesia [67–69]. In our review, the ALFF value of the insula increased after acupuncture treatment, suggesting that acupuncture treatment of FC may be related to regulating the functional activity of the insula and affecting the conduction of gastrointestinal signals [30].The thalamus is a relay station for sensory nerves, receiving sensory information from various parts of the body and projecting to the cortex [70]. The results of this study showed that brain activation in the thalamus and insula increased during visceral dilation in IBS patients after acupuncture treatment [28]. This implies that the abnormal excitation of the insula and thalamus could be caused by visceral sensitivity and psychological state, and acupuncture could modulate the abnormal excitation of related brain regions.

The HIPP is an important part of the limbic system, which is involved in the formation of memory and emotion. It is an important emotion regulation center and has always been an important nucleus in the study of depressive [71]. The paraHIPP is the main cortical input to the HIPP and is involved in the regulation of emotion during visceral responses [72, 73]. Multiple fMRI studies have confirmed that the functional activity of the HIPP and paraHIPP in patients with FGIDs is different from that in healthy individuals [74–76]. The comprehensive results of our systematic review showed that, the ALFF value of the HIPP and paraHIPP of FGIDs patients increased after acupuncture treatment [23, 25, 30], and Ma’s research showed that the functional connectivity of the left HIPP and bilateral insula was enhanced in patients with FC [32]. The amygdala is also one of the components of the limbic system, an important part of the emotional network and considered to be an important brain structure responsible for generating and regulating emotions [77, 78]. The amygdala is essential for processing visceral afferent and efferent signals, anticipatory inhibition of visceral pain, and emotional arousal response [13, 79]. fMRI study has found that BLA (the primary sensory input region of the amygdala) is a main amygdala subregion showing abnormal functional connectivity in FD compared to healthy subjects [80]. One article in our study also confirmed extensive cerebral changes in the BLA rsFC in the obvious *Deqi* group after acupuncture treatment. In addition, the efficacy advantage of the acupuncture *Deqi* group was related to the influence of BLA rsFC [21].

**Fig. 5:**
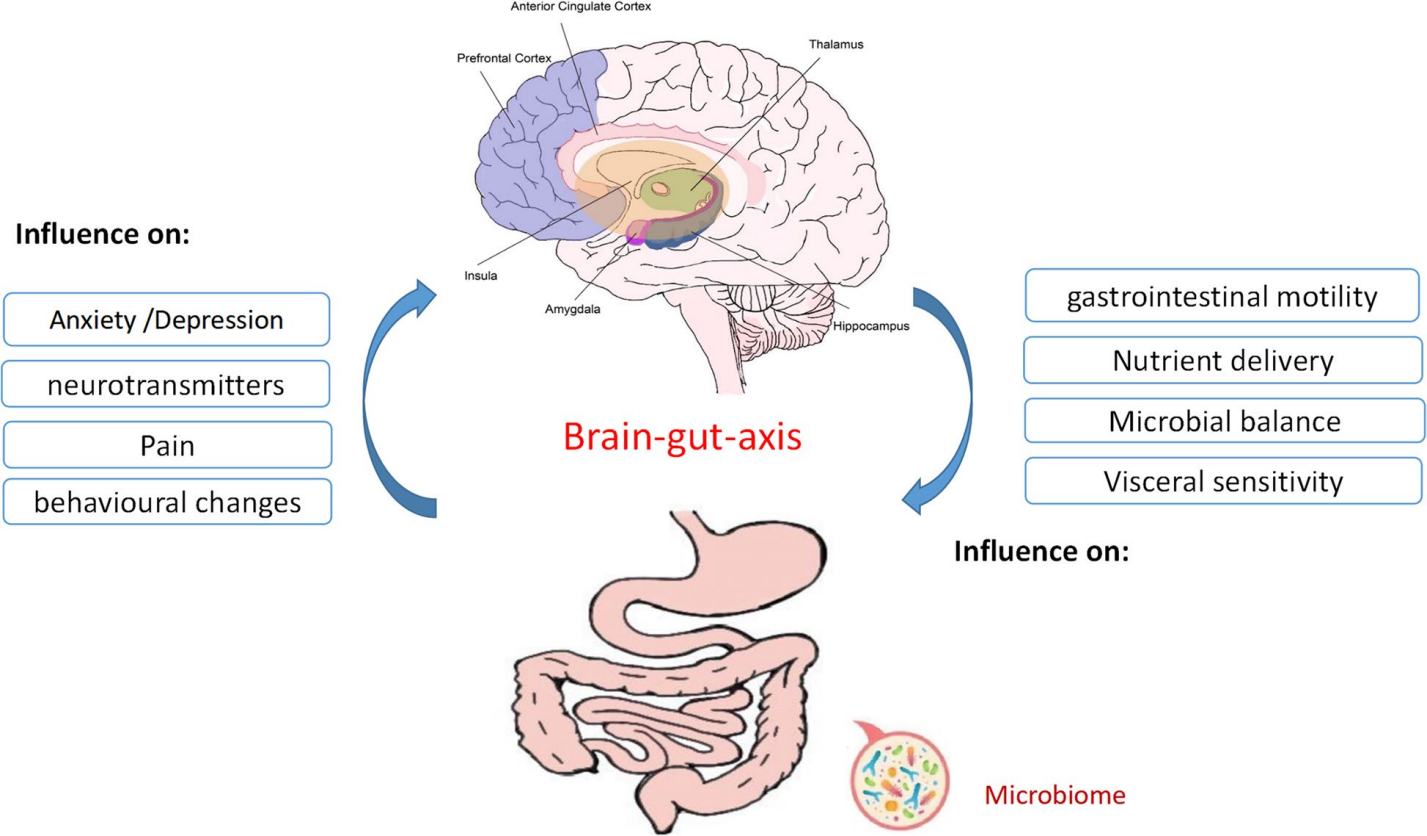
Correlation between brain-gut axis and functional gastrointestinal disease

Anxiety and depression lead to a double acceleration in the incidence of gastrointestinal symptoms [81]. In this systematic review, five studies assessed the psychological state of patients with FGIDs and reported significant decreases in scores after both acupuncture and sham acupuncture/moxibustion treatment [21, 23, 24, 26, 27, 29]. Only one of these studies reported that the improvement of anxiety and depression scores in IBS-C patients after acupuncture treatment was significantly higher than that in the moxibustion group [27]. This suggests that more attention should be paid to the examination and management of neuropsychiatric symptoms in patients with FGIDs, and acupuncture therapy can be used as a complementary and alternative therapy to improve their symptoms. For patients with FGIDs, abnormal central activity due to gastrointestinal dysfunction (bottom-up) and gastrointestinal problems due to brain dysfunction (top-down) occur, suggesting that the brain-gut axis mediates bidirectional information communication between the periphery and the brain (Fig. 5). On the basis of our results, acupuncture can regulate the functional changes in the brain regions related to FGIDs symptoms, and improve the corresponding clinical symptoms, visceral sensation and psychological state, and its specific mechanism may be related to promoting the functional normalization of brain regions related to brain-gut axis and restoring gastrointestinal homeostasis. But the results of our systematic review is insufficient to support whether the acupuncture treatment is effective only for symptom relief or their underlying causes.

### Strengths and limitations

Strengths:1)Our study is the first to summarize and evaluate relevant clinical controlled trials, aiming to conduct a comprehensive investigation of the core brain regions related to acupuncture treatment of FGIDs.2)Acupuncture can improve the symptoms and quality of life of FGIDs.

limitations: 1) Since this study included rigorous randomized controlled trials, including three types of FGIDs, the number of studies in each subgroup was limited, so the study may be biased. 2) Heterogeneity of the included trials. Given the different disease types, interventions, acupoint protocols, clinical outcomes, and assessment methods in these studies, it is difficult to conduct further meta-analyses of all relevant data. 3) At present, the research methods on the central mechanism of FGIDs are relatively single. Among the 10 included studies, only 3 studies on IBS used task-state fMRI, and the remaining 7 studies all used resting-state fMRI. Although the analysis of image data is relatively abundant, including ALFF, ReHo, and FC analysis, if multimodal image analysis can be used, the central mechanism characteristics of FGIDs can be explored more comprehensively. 4) Lack of high-quality methodological studies. Flaws in implementation or reporting, such as allocation concealment, blinding, and outcome assessment, may reduce the confidence of the study results. Therefore, future related research should pay more attention to the shortcomings of the above research design and methodology, in order to improve the reliability of the research results of the central mechanism of FGIDS patients. 5) Although our study results show that acupuncture can improve the symptoms of FGIDs patients, it cannot rule out spontaneous relief of symptoms and the potential placebo effect of acupuncture.

## Conclusion

The Clinical data results showed that acupuncture (MA, EA, and taVNS) treatment could regulate the functional connectivity and activity of brain regions such as insula, ACC, PFC, thalamus, HIPP, and amygdala in patients with FGIDs, improved the clinical symptoms of FGIDs patients, and could relieve anxiety and depression symptoms of patients. But our current systematic review is insufficient to recommend acupuncture as a first-line treatment method and to determine the long-term results of acupuncture treatment.

### Supplementary Information

**Additional file 1.**

## Data Availability

The data supporting this research article are available from the corresponding author on reasonable request.
