# Supplementary material for: Symptom effects and central mechanism of acupuncture in patients with functional gastrointestinal disorders: a systematic review based on fMRI studies
Source: BMC Gastroenterol. 2024 Jan 24;24:47. doi: 10.1186/s12876-024-03124-y (PMC10809475; doi:10.1186/s12876-024-03124-y)
Supplement: Supplementary file 1 — Additional file 1. [file 12876_2024_3124_MOESM1_ESM.docx]

**The specific search strategies applicable to PubMed are as follows:**

**#1 (((((((((((((((((((((((((Gastrointestinal Diseases[MeSH Terms]) OR (Disease, Gastrointestinal[Title/Abstract])) OR (Diseases, Gastrointestinal[Title/Abstract])) OR (Gastrointestinal Disease[Title/Abstract])) OR (Gastrointestinal Disorders[Title/Abstract])) OR (Gastrointestinal Disorder[Title/Abstract])) OR (Functional Gastrointestinal Disorders[Title/Abstract])) OR (Functional Gastrointestinal Disorder[Title/Abstract])) OR (Gastrointestinal Disorder, Functional[Title/Abstract])) OR (Gastrointestinal Disorders, Functional[Title/Abstract])) OR (Cholera Infantum[Title/Abstract])) OR (Functional dyspepsia[Title/Abstract])) OR (postprandial distress syndrome[Title/Abstract])) OR (epigastric pain syndrome[Title/Abstract])) OR (Irritable Bowel Syndrome[MeSH Terms])) OR (Irritable Bowel Syndromes[Title/Abstract])) OR (Syndrome, Irritable Bowel[Title/Abstract])) OR (Syndromes, Irritable Bowel[Title/Abstract])) OR (Colon, Irritable[Title/Abstract])) OR (Irritable Colon[Title/Abstract])) OR (Colitis, Mucous[Title/Abstract])) OR (Colitides, Mucous[Title/Abstract])) OR (Mucous Colitides[Title/Abstract])) OR (Mucous Colitis[Title/Abstract])) OR (Functional constipation[Title/Abstract])) OR (chronic functional constipation[Title/Abstract])**

**#2 (Acupuncture[MeSH Terms]) OR (Pharmacopuncture[Title/Abstract])**

**#3 ((((((((((((((((((((((((((((((((((((((((Magnetic Resonance Imaging[MeSH Terms]) OR (Imaging, Magnetic Resonance[Title/Abstract])) OR (NMR Imaging[Title/Abstract])) OR (Imaging, NMR[Title/Abstract])) OR (Tomography, NMR[Title/Abstract])) OR (Tomography, MR[Title/Abstract])) OR (MR Tomography[Title/Abstract])) OR (NMR Tomography[Title/Abstract])) OR (Steady-State Free Precession MR[Title/Abstract])) OR (Steady State Free Precession MRI[Title/Abstract])) OR (Zeugmatography[Title/Abstract])) OR (Imaging, Chemical Shift[Title/Abstract])) OR (Chemical Shift Imagings[Title/Abstract])) OR (Imagings, Chemical Shift[Title/Abstract])) OR (Shift Imaging, Chemical[Title/Abstract])) OR (Shift Imagings, Chemical[Title/Abstract])) OR (Chemical Shift Imaging[Title/Abstract])) OR (Magnetic Resonance Image[Title/Abstract])) OR (Image, Magnetic Resonance[Title/Abstract])) OR (Magnetic Resonance Images[Title/Abstract])) OR (Resonance Image, Magnetic[Title/Abstract])) OR (Magnetization Transfer Contrast Imaging[Title/Abstract])) OR (MRI Scans[Title/Abstract])) OR (MRI Scan[Title/Abstract])) OR (Scan, MRI[Title/Abstract])) OR (Scans, MRI[Title/Abstract])) OR (Tomography, Proton Spin[Title/Abstract])) OR (Proton Spin Tomography[Title/Abstract])) OR (fMRI[Title/Abstract])) OR (MRI, Functional[Title/Abstract])) OR (Functional MRI[Title/Abstract])) OR (Functional MRIs[Title/Abstract])) OR (MRIs, Functional[Title/Abstract])) OR (Functional Magnetic Resonance Imaging[Title/Abstract])) OR (Magnetic Resonance Imaging, Functional[Title/Abstract])) OR (Spin Echo Imaging[Title/Abstract])) OR (Echo Imaging, Spin[Title/Abstract])) OR (Echo Imagings, Spin[Title/Abstract])) OR (Imaging, Spin Echo[Title/Abstract])) OR (Imagings, Spin Echo[Title/Abstract])) OR (Spin Echo Imagings[Title/Abstract])**

**#4 (Randomized controlled trial[MeSH Terms]) OR (Randomized[Title/Abstract])**

**#1 AND #2 AND #3 AND #4**
